# Incorporating longitudinal history of risk factors into atherosclerotic cardiovascular disease risk prediction using deep learning

**DOI:** 10.21203/rs.3.rs-3405388/v1

**Published:** 2023-10-13

**Authors:** Jingzhi Yu, Xiaoyun Yang, Yu Deng, Amy E. Krefman, Lindsay R. Pool, Lihui Zhao, Xinlei Mi, Hongyan Ning, John Wilkins, Donald M. Lloyd-Jones, Lucia C. Petito, Norrina B. Allen

**Affiliations:** Northwestern University; Northwestern University; Northwestern University; Northwestern University; Northwestern University; Northwestern University; Northwestern University; Northwestern University; Northwestern University; Northwestern University; Northwestern University; Northwestern University

**Keywords:** Risk Prediction, Longitudinal Data, Cardiovascular Disease, Deep Learning, Clinical Guidelines

## Abstract

**Background:**

It is increasingly clear that longitudinal risk factor levels and trajectories are related to risk for atherosclerotic cardiovascular disease (ASCVD) above and beyond single measures. Currently used in clinical care, the Pooled Cohort Equations (PCE) are based on regression methods that predict ASCVD risk based on cross-sectional risk factor levels. Deep learning (DL) models have been developed to incorporate longitudinal data for risk prediction but its benefit for ASCVD risk prediction relative to the traditional Pooled Cohort Equations (PCE) remain unknown.

**Objective:**

To develop a ASCVD risk prediction model that incorporates longitudinal risk factors using deep learning.

**Methods:**

Our study included 15,565 participants from four cardiovascular disease cohorts free of baseline ASCVD who were followed for adjudicated ASCVD. Ten-year ASCVD risk was calculated in the training set using our benchmark, the PCE, and a longitudinal DL model, Dynamic-DeepHit. Predictors included those incorporated in the PCE: sex, race, age, total cholesterol, high density lipid cholesterol, systolic and diastolic blood pressure, diabetes, hypertension treatment and smoking. The discrimination and calibration performance of the two models were evaluated in an overall hold-out testing dataset.

**Results:**

Of the 15,565 participants in our dataset, 2,170 (13.9%) developed ASCVD. The performance of the longitudinal DL model that incorporated 8 years of longitudinal risk factor data improved upon that of the PCE [AUROC: 0.815 (CI: 0.782–0.844) vs 0.792 (CI: 0.760–0.825)] and the net reclassification index was 0.385. The brier score for the DL model was 0.0514 compared with 0.0542 in the PCE.

**Conclusion:**

Incorporating longitudinal risk factors in ASCVD risk prediction using DL can improve model discrimination and calibration.

## Introduction

The Pooled Cohort Equations (PCE) were developed by the American College of Cardiology (ACC) and American Heart Association (AHA) in 2013 and updated in 2018 using data from 9 longitudinal cohort studies as a tool for clinicians to predict 10-year risk of atherosclerotic cardiovascular disease (ASCVD).^1, 2^ The PCE are a set of race- and sex-specific Cox proportional hazards models, that include widely-accepted clinical and behavioral risk factors for ASCVD, including age, sex, race, systolic (SBP) and diastolic blood pressure (DBP), total cholesterol, high density lipid-protein (HDL) cholesterol, smoking status, and type 2 diabetes. In clinical practice, risk predictions from the PCE are a key criterion to determine eligibility for moderate to high intensity statins and hypertension treatments.^1, 3^ However, numerous studies have found the performance of the PCE varies across demographic groups^4–6^; c-statistics from these studies ranged from 0.55 to 0.77 (average: 0.70) in men and 0.61 to 0.82 (average: 0.74) in women.^7, 8^ Additionally, current clinical guidelines provide more ambivalent and complex treatment recommendations for those who fall in the borderline (5–7.5%) and intermediate risk groups (7.5–20%).^9^ A more accurate and robust risk prediction algorithm can help physicians better assess an individual’s risk, allowing them to make more appropriate treatment decisions.

A growing number of studies have demonstrated that long-term risk factor levels are associated with an individual’s risk for the development of ASCVD. For instance, incident CVD risk was shown to be dependent on cumulative exposure to LDL-C.^10^ In a separate study, incident CVD and survival were also found to be associated with 10-year cumulative SBP.^11^ Hence, long-term risk factor patterns may be predictive of ASCVD risk above and beyond cross-sectional levels.^12^ In a prior study, after including 5-year and 10-year cumulative blood pressure measurements in the PCE, researchers found a moderate improvement in the net reclassification index.^13^ Additionally, full integration of multiple longitudinal trajectories of clinical factors into ASCVD prediction is now feasible in clinical practice given advances in computing and electronic medical record (EMR) systems that allow clinicians to access longitudinal risk factor data for their patients.

In recent years, deep learning methods have been applied to many clinical predictive and classification problems to much success.^14–16^ Compared with traditional statistical methods, deep learning methods are often superior at processing and creating representations of complex data, such as radiology images and unstructured physician notes,^17, 18^ without the need of prior feature engineering or selection.^15, 19^ Hence, deep learning can more thoroughly extract and leverage the rich features stored in longitudinal data such as longitudinal blood pressure measurements recorded in the electronic health records (EHR) for predictive tasks.

In this study, we incorporated cross-sectional and longitudinal clinical and behavioral risk factor levels into a state-of-the-art deep learning architecture to create a new prediction model for 10-year risk of incident ASCVD in a pooled cohort of 4 US-based, diverse longitudinal cohorts. We evaluated our model’s predictive performance in comparison to that of the PCE in the overall population and in key population subgroups to better understand the importance of longitudinal data for ASCVD risk prediction. Moreover, we determined the importance of each clinical variable used in the prediction model. Lastly, we performed additional evaluations of the model performance in the borderline and intermediate risk groups to better understand our model’s potential impact on clinical decision making.

## Results

### Baseline Characteristics

Baseline demographics and measurements of CVD risk factors included in the PCE are described in Table 1. Pooled cohort participants included in this study were 55% female, 27% non-Hispanic Black and 50 years old on average. We found participants who developed ASCVD in prediction period had significantly higher levels of ASCVD risk factors compared with the participants who did not develop ASCVD.

### Performance of Models

Table 3 shows the discrimination of the three models in the training and testing datasets. The AUCs for the PCE and the longitudinal *Dynamic-DeepHit* model were 0.792 (CI: 0.760–0.825) and 0.815 (CI: 0.782–0.844), respectively. The *Dynamic-DeepHit* model shows slight improvement in discrimination upon the PCE model. The cross-sectional deep learning model achieved an AUC of 0.807 (CI: 0.778–0.838) (**Supplemental Table 2**). The continuous net reclassification index (NRI) for the *Dynamic-DeepHit* model compared with the PCE was 0.385. The Brier Score for the PCE model was 0.054, 0.052 for the cross-sectional deep learning model and 0.051 for the longitudinal deep learning model, showing meaningful improvement in model calibration.

The predicted risks derived from the *Dynamic-DeepHit* model were found to be generally lower than the risks derived from the PCE (Figure 2). In Figure 3, the calibration of the *Dynamic-DeepHit* model is compared with the calibration of the PCE by comparing the predicted risk and observed risk within each decile of predicted risk. The PCE is shown to over-predict 10-year ASCVD risk, especially within the top 40% of predicted risk, which corresponds to the 7.5% risk threshold used in clinical guidelines. Comparatively, the calibration of the *Dynamic-DeepHit* model is consistently better along the entire spectrum of risk.

### Model Performance in Population Subgroups

The discriminative performance of the *Dynamic-DeepHit* and PCE models within different population groups is shown in Table 3. The *Dynamic-DeepHit* model performed relatively better than the PCE in the other-males group (0.801, CI: 0.753–0.848 vs 0.779, CI: 0.732–0.826), other-females group (0.801, CI : 0.737–0.764 vs 0.780, CI : 0.712–0.848), and the Black-females group (0.821, CI : 0.751–0.890 vs 0.801, CI 0.726–0.877). However, it underperformed in the Black-males group (0.820, CI : 0.751–0.888 vs 0.826, CI : 0.756–0.897). In the under-60-years-old group, the *Dynamic-DeepHit* modelhad an AUROC of 0.803 (CI: 0.747–0.858) compared with the PCE’s AUROC of 0.781 (CI: 0.721–0.842) and in the over-60-years-old group, the *Dynamic-DeepHit* model had an AUROC of 0.698 (CI: 0.646–0.749) compared with the PCE’s AUROC of 0.667 (0.615–0.719). The *Dynamic-DeepHit* model outperforms the PCE in three of the four demographic groups outlined by the PCE.

### Feature Importance

The results of the leave-one-out feature importance analysis are shown in Figure 4. After removing age from the model, the greatest decrease in AUROC was observed (0.769, CI: 0.735–0.803); thus, age is considered the most important variable in the model. Following age, longitudinal SBP was the second most important predictor, with the AUROC reduced to 0.777 (CI: 0.744–0.809). Diabetes diagnosis and hypertension treatment were the most important categorical predictors, with AUROCs reduced to 0.779 (CI: 0.747–0.812) and 0.780 (CI: 0.748–0.813) when these predictors were removed respectively.

Figure 5 shows the longitudinal trajectories of clinical risk factors, including SBP, DBP, total cholesterol and HDL among the individuals whose risk increased and those whose risk decreased after switching to the *Dynamic-DeepHit* model for ASCVD risk prediction. Between the two groups, the average terminal measurements of SBP and total cholesterol were similar, the historical measurements of those risk factors were higher among those whose predicted risk increased in *Dynamic-DeepHit* model.

### Borderline Risk Stratification

Among the individuals in the borderline and intermediate risk groups determined by the risk derived from the PCE, the AUC from the *Dynamic-DeepHit* model was higher than that from the PCE: 0.688 (CI: 0.634–0.742) versus 0.652 (CI: 0.594–0.709). The NRI for the *Dynamic-DeepHit* model between the borderline and intermediate group was 0.322. The Brier score was 0.069 for the PCE compared with 0.067 for the *Dynamic-DeepHit* model, again showing some improvement in the model calibration.

Given the 7.5% risk threshold for moderate-intensity statin prescription, we examined the individuals whose risk crossed the threshold in both directions to understand the *Dynamic-DeepHit* model’s potential impact on clinical decision making. In our testing dataset, among those who would be prescribed statins under the PCE risk (N=1,213), 33% (N=405) would not be prescribed statins under the new risk provided by the *Dynamic-DeepHit* model, and 95% (N=386) of those individuals would not develop ASCVD. Among those who were not prescribed statins using the PCE (N=1,900), 2% (N=34) would be recommended to prescribe statins under the *Dynamic-DeepHit* model. However, of those individuals, only 3% (N=1) developed ASCVD within 10 years.

## Discussion

### Principal Findings

In this study, we have demonstrated that by incorporating longitudinal data of the same clinical and behavioral predictors as in the PCE using a state-of-the-art and validated deep learning model we can improve the calibration of predicting 10-year ASCVD risk. We leveraged data from 4 diverse cohorts for model training and testing and found that the longitudinal deep learning model outperformed the PCE both in the overall cohort and in specific subpopulations. We have demonstrated that the longitudinal deep learning model has clinical value through improved discrimination and greater calibration for those with borderline risk of ASCVD, thus providing physicians more reliable estimates of risk for clinical decision making.

### Deep Learning in ASCVD Risk Prediction

Longitudinal trends of clinical factors such as blood pressure and cholesterol have long been established to be of clinical importance.^13^ While this is not the first study to incorporate longitudinal data for predicting ASCVD, to our knowledge, it is the first study that uses a deep learning approach. Prior studies used methods such as including aggregate summary statistics of the longitudinal clinical data in the PCE or landmark models that could update data at fixed time intervals.^13, 25, 26^ This foundational work led to minor improvements in model discrimination; however, we were able to achieve better performance because we utilized a deep learning method. A key advantage of deep learning models is their ability to recognize complex patterns by utilizing multiple layers of artificial neural networks, which are composed of inter-connected nodes. This advantage manifests in two ways in the *Dynamic-DeepHit* model. First, the improvement in the discrimination of the cross-sectional *DeepHit* model over the PCE demonstrates that given the same cross-sectional data, neural networks can make better predictions of ASCVD than the PCE. Second, the RNN can create robust representations of longitudinal clinical data in the presence of missing values, preserving critical information for ASCVD risk prediction.

### Clinical Implications

Through evaluating the *Dynamic-DeepHit* model in various population demographic groups, we found that the model improves risk prediction in Black females compared with the PCE.^27–32^ The *Dynamic-DeepHit* model performance in Black females may indicate that incorporating longitudinal data can better elucidate higher risks, allowing physicians to make more accurate treatment decisions and reducing health outcome disparities in these high-risk groups.

Among the individuals categorized as borderline- and intermediate-risk by the PCE, the *Dynamic-DeepHit* model improved discrimination and was better calibrated. One-third of the individuals in the intermediate PCE risk groups had overestimated 10-year ASCVD risk, which indicates the potential for over-prescribing. In these individuals, the *Dynamic-DeepHit* model slightly under-estimates risk, that it is better at ruling out people who will not have ASCVD events, while not as good as identifying those who will get ASCVD. As current clinical guidance requires further risk analysis for the individuals in these risk groups, guideline-concordant treatment is less optimal. By providing a better calibrated risk assessment, clinicians may be less concerned with over-prescribing and feel more confident in prescribing guideline-concordant treatment given the predicted risks from the *Dynamic-DeepHit* model.

The feature importance analysis shows that longitudinal measurements of clinical variables have meaningful influence on the performance on the *Dynamic-DeepHit* model. In the *Dynamic-DeepHit* model, longitudinal SBP was the most important modifiable predictor, while total cholesterol was found to be relatively important as well. Similar to prior research^22^, age was found to be an important predictor in the *Dynamic-DeepHit* model. In addition, diabetic status, sex, and smoking status were also found to influence the AUROC of the model. In the observed 8-year trajectories of SBP, DBP, and total cholesterol, for the individuals whose risk changed (Figure 5), at the population level, the aggregate terminal measurements were similar. If prediction occurred only using those terminal measurements, a similar risk profile between those with increased risk and decreased risk would be assumed. However, the Dynamic-DeepHit model picked up separation in the historical values of those clinical factors, which contributed to the model identifying the differences in risk profiles of the two groups of individuals. Combined with the results of the feature importance analysis, this evidence further supports that longitudinal histories of clinical predictors can provide additional insight in evaluating ASCVD risk profiles.

With the proliferation of EHRs, longitudinal data is readily accessible. In addition, with the advent of cloud and edge computing, it is possible to deliver intensive computing capabilities to the EHR for supporting sophisticated machine learning or deep learning models for clinical risk prediction. This study shows, with further validation, deep learning models can be a powerful tool to aid clinicians to leverage the silos of currently untapped historical patient data in the EHR to improve patient cardiovascular outcomes. New methods of interpreting these models will also add confidence in adoption among physicians.

### Limitations

There are several limitations in our study. First, the cohorts used in this study may not reflect the clinical conditions of present-day patients, who are more likely to be on CVD treatments, such as statins. Therefore, given the limited information we had on statin usage, we did not exclude any participants who may have been on statin treatment. Second, data was recorded more sparsely in the cohort studies, whereas clinical measurements are often more frequent in clinical practice^33^. The quality of the data stored in the EHR could be also compromising, due to varying clinical contexts of when the data was collected. While these data problems exist, deep learning methods are still one of the best tools to overcome such issues. ^34, 35^ On the other hand, EHRs often do not contain up to 8 years of longitudinal data on patients. As this study is a proof of concept, further work is needed to explore efficacy and utility of incorporating longitudinal risk factors into ASCVD risk prediction within EHRs.

## Methods

### Study Population

The four longitudinal cohorts used in this study contributed data to the Cardiovascular Lifetime Risk Pooling Project (LRPP): the Framingham Heart Study, Framingham Offspring Study, Coronary Artery Risk Development in Young Adults (CARDIA) Study, and Atherosclerosis Risk in Communities (ARIC) Study.^20^ These cohorts were selected for their number of participants, duration of follow-up, number of participant visits, and consistency of measurement of CVH risk factors.

**Figure 1:**
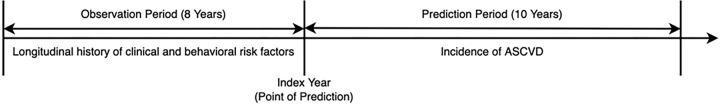
Timeframe of the study. The longitudinal history of clinical and behavior risk factors within the 8 years prior to the index year were included in our prediction model. Our prediction model predicts the outcome within 10 years after the index year.

**Figure 2:**
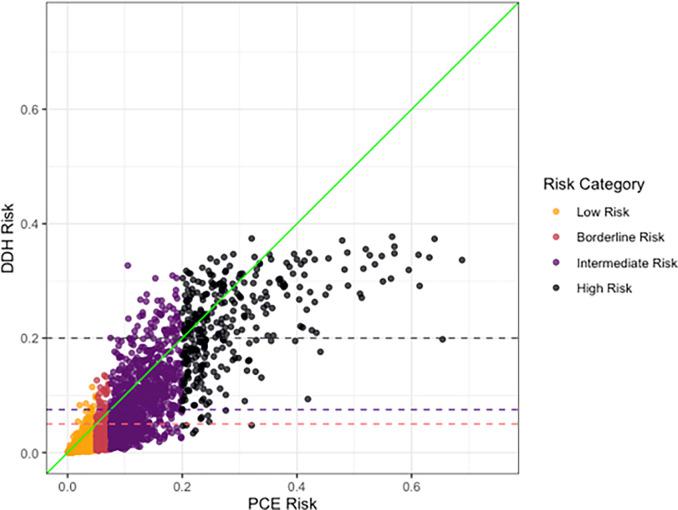
Predicted risk (actual value) comparison between two models. Risk category derived from PCE risk category thresholds. horizontal dashed lines show the numeric thresholds for the risk categories used by the PCE mapped on to the risk predicted by the *Dynamic-DeepHit* model.

**Figure 3:**
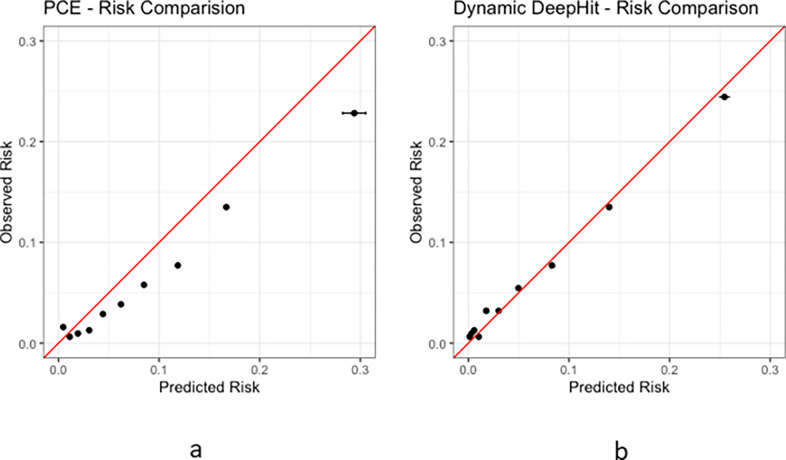
Observed events compared with average predicted risk within each predicted risk decile. Confidence intervals are also shown via error bars. **a)** Predicted vs observed risk in PCE **b)** Predicted vs observed risk in *Dynamic-DeepHit*

**Figure 4:**
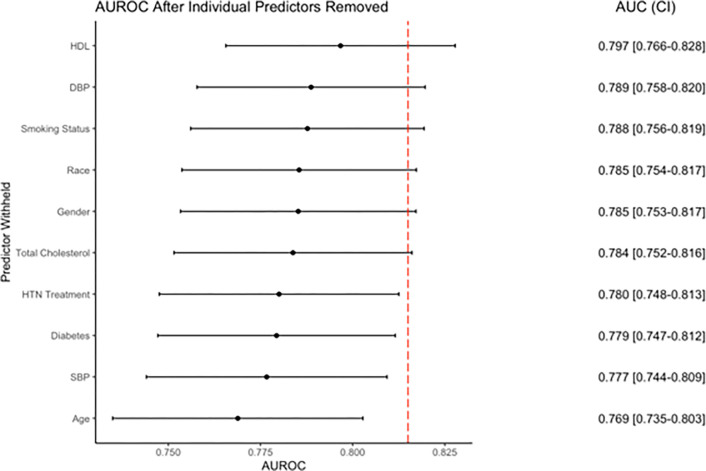
Feature Importance. AUROC was calculated via retraining and retesting the model while removing predictors individually. Red dashed line indicates the AUROC of the full *Dynamic-DeepHit* model.

**Figure 5:**
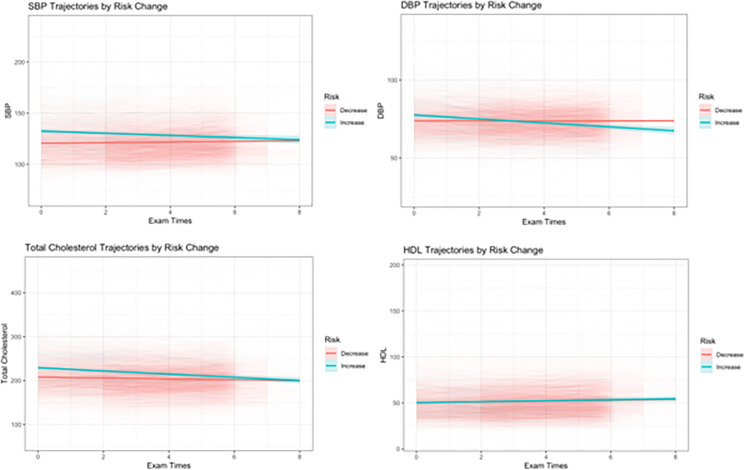
Longitudinal histories of clinical risk factors compared between individuals whose risk increased and decreased in the *Dynamic-DeepHit*model from the PCE. Generalized linear models were used to smooth the longitudinal clinical risk factor trajectories. Color bands show the 95% confidence interval.

As the examination schedules differed across cohorts, the number of exams within timeframes varied. To include the largest number of exams across the different studies while balancing the size of the timeframe for the study, we used 8 years of longitudinal data as the timeframe for CVD risk factor ascertainment (observation period). For consistency with the PCEs, outcomes were then measured over a 10-year follow-up period. Thus, to maximize the number of exams included in our study, we included data beginning at the following index exams (i.e. the exam at which risk factor follow-up began) for the included studies (Figure 1): year 15 for the Framingham Heart Study, year 10 for the Framingham Offspring Study, year 18 for the CARDIA study, and year 1 for the ARIC study. The exact start and end years of each cohort as well as their mean and interquartile range of the number of exams in each cohort are shown in Table 2.

Eligible participants were over 40 and under the age of 75 years at the point of prediction (i.e. the end of the 8 year observation period), had no record of self-report or diagnosed ASCVD at the index exam or during the 8 year observation period, and had at least one measurement of SBP, DBP, total cholesterol and HDL cholesterol. The LRPP is approved by the Northwestern IRB and this study utilized de-identified data from each of the included cohorts in LRPP. Written informed consent was obtained for all participants and analysis were performed in accordance with relevant guidelines.

### Outcome: ASCVD incidence

The outcome in our study was ASCVD incidence, defined as the incidence of coronary heart disease, ischemic stroke, or CVD-related death, over a 10-year period that began at the end of the observation period (Figure 1).^11, 20^ Coronary heart disease and ischemic stroke were adjudicated by review of medical records by study investigators.^21^ Participants without any recorded event at the end of the study, or who died of other causes during the follow-up period were considered right censored.

### Features: CVD Risk Factors

CVD risk factors included in the original PCE include systolic BP, diastolic BP, total cholesterol, and HDL cholesterol, and were measured 1–4 times during the 8-year observation period. Blood pressure was measured using standard methods by clinic staff in the various cohorts.^21, 22^ Fasting HDL-C, total cholesterol measurements and blood glucose were collected via blood serum.^20, 22^ Diagnosis of diabetes and treatment for hypertension, predictors also included in the PCE, were self-reported at the index visit.^21, 22^ Age, sex, race, ethnicity, smoking status, and alcohol consumption were self-reported at the index visit.^21, 22^

### Statistical Analysis

The deep learning model used in this study is *Dynamic-DeepHit*, which enabled the incorporation of longitudinal risk factor data in a dynamic fashion to estimate 10-year risk of incident ASCVD.^23^ The *Dynamic-DeepHit* model has been demonstrated to have substantial improvements over traditional predictive methods, including the Cox Proportional Hazards Model, in predicting cystic fibrosis outcomes.^23^

The *Dynamic-DeepHit* model consists of two neural networks: 1) a recurrent neural network (RNN) that processes the longitudinal measurements and predicts future measurements of time-varying covariates, and 2) a fully connected neural network that estimates the probability of the specific event at a given time. RNNs are commonly used for machine learning problems involving temporal or sequential data and can capture long-term dependencies in the data. The *Dynamic-DeepHit* model also utilizes an attention mechanism that identifies important longitudinal measurements when making risk predictions, which improves predictive performance. The second neural network takes as input the learned representations that are output from the first neural network along with the last recorded set of behavioral and clinical covariates (e.g. the most recent CVD risk factor measurements at the end of the 8-year observation period). The output layer of the second neural network converts the learned relationships between the risk factors and outcome into the 10-year risk of incident CVD.

To explore the reasons for any improvements in the predictive power we also implemented a cross-sectional *DeepHit* model. This allowed us to disentangle whether the improvements were due to the incorporation of the longitudinal data or simply to the complexity of the neural network modeling methods. The *DeepHit* model was fitted on only the last set of measurements for each participant within the 8-year observation period. We also fit the traditional PCE model, to understand its performance in this sample.

Data pre-processing included randomly splitting the dataset into 3 chunks, called training, tuning, and testing, at a 3:1:1 ratio. The *Dynamic-DeepHit* and cross-sectional *DeepHit* models were trained in the training dataset and corresponding hyperparameters were tuned in the tuning dataset. The training data for the PCE included both the training and tuning datasets. The testing dataset, not used in model development, was used for validation. The participants were the same in each of the respective datasets for each model.

We assessed model discrimination and calibration of all 3 models. We calculated and compared the Area Under the Receiver Operator Curve (AUROC) for all models to evaluate model discrimination, the ability of the model to discriminate those who have a higher risk of having an event from those at lower risk. Brier scores were used to evaluate the calibration of the model; lower scores indicate better calibration, the extent of the estimated risk correspond to observed event rates.^24^

The trained *Dynamic-DeepHit* model was evaluated in the following population groups: Black males, Black females, other (White, Hispanic, Asian) males, other females, under 60 years old and 60 or over years old. These demographic groups were chosen to mirror the same classifications used for the sex- and race-specific PCE. As in the overall analysis, the AUROCs were compared between corresponding population subgroups.

To understand the importance of each predictor in the *Dynamic-DeepHit* model, we took a leave-one-out approach. We removed one predictor at a time from the *Dynamic-DeepHit* model and retrained and retested the model. The change in the testing dataset AUROC was calculated for each feature removed: the greater the change in AUROC, the greater the importance of the predictor. To also understand the role of longitudinal clinical risk factors better in the *Dynamic-DeepHit* model, we examined the average trajectories of SBP, DBP, total cholesterol and HDL for the individuals whose predicted risk increased and those whose risk decreased in the *Dynamic-DeepHit* model. Trajectories were created via generalized estimating equations (GEE) to account for correlation between repeated measurements for individuals. The trajectories were visualized across exam times with the 95% confidence bands.

Current blood pressure and cholesterol control guidelines use risk thresholds based on the PCE to inform clinical care. Physicians are advised to prescribe medium intensity statins if an individual’s ASCVD risk is over 7.5%. However, differentiation of individuals between the borderline and intermediate PCE risk groups could be improved. We calculated the net reclassification index (NRI) between the PCE and the *Dynamic-DeepHit model,* to understand how the Dynamic-DeepHit model changed individuals’ risk classification. We then conducted additional analysis to better understand the performance of the *Dynamic-DeepHit* model in borderline and intermediate groups, and how clinical behavior would be affected if the risk derived from the *Dynamic-DeepHit* model was used instead of risk from the PCE.

All statistical analysis was performed using Python version 3.8 and R 4.0.2. A 5% type-I error rate was used when calculating all confidence intervals.

## Figures and Tables

**Table 1.** Baseline demographics of LRPP analytic dataset. Risk factors in the ASCVD group were significantly higher than those in the Non-ASCVD group.

Variables	Overall	Non-ASCVD	ASCVD
	*(N=15565)*	*(N=13395)*	*(N=2170)*
Age (Mean, SD)	50.2 (7.2)	49.6 (7.0)	53.7 (6.9)
Sex			
Male	7028 (45.2%)	5798 (43.3%)	1230 (56.7%)
Female	8537 (54.8%)	7597 (56.7%)	940 (43.3%)
Race			
Other	11366 (73.0%)	9750 (72.8%)	1616 (74.5%)
Black	4199 (27.0%)	3645 (27.2%)	554 (25.5%)
Systolic Blood Pressure, mmHg	121 (18.7)	120 (17.6)	131 (21.9)
Diastolic Blood Pressure, mmHg	74.8 (11.6)	74.1 (11.2)	78.9 (12.9)
Total Cholesterol, mg/dL	207 (42.9)	205 (41.6)	223 (47.3)
High Density Lipid, mg/dL	51.6 (16.5)	52.4 (16.5)	46.9 (15.6)
Smoker (vs. Never Smoker)	4623 (29.7%)	3752 (28.0%)	871 (40.1%)
Diabetes	1410 (9.1%)	966 (7.2%)	444 (20.5%)
Treated for Hypertension	3726 (23.9%)	2946 (22.0%)	780 (35.9%)

**Table 2.** The official start year, start year of the observation period (after adjustment), end year of the 8 year follow-up period, average number of exams within the 8 year follow-up period as well as the interquartile range of the number of exams by each cohort.

Cohort	Official Start Year	Start Year of Observation Period	End Year of Observation Period	End of Follow-up	Average Number of Exams	Interquartile Range
ARIC	1987	1987	1995	2005	3	3–3
CARDIA	1985	2003	2011	2021	2	2–2
FHS	1948	1963	1971	1981	2	1–3
FOF	1971	1981	1989	1999	2	2–2

**Table 3.** Discrimination performance of models and in population subgroups.

Categories	PCE Training AUROC	*Dynamic-DeepHit* Training AUROC	PCE Testing AUROC	*Dynamic-DeepHit* Testing AUROC
Full Model	0.801 (0.786–0.816)	0.817 (0.791–0.843)	0.792 (0.760–0.825)	0.815 (0.782–0.844)
Race & Sex				
Other Males	N/A	N/A	0.779 (0.732–0.826)	0.801 (0.753–0.848)
Other Females	N/A	N/A	0.780 (0.712–0.848)	0.801 (0.737–0.864)
Black Males	N/A	N/A	0.826 (0.756–0.897)	0.820 (0.751–0.888)
Black Females	N/A	N/A	0.801 (0.726–0.877)	0.821 (0.751–0.890)
Age				
< 60 years	N/A	N/A	0.781 (0.721–0.842)	0.803 (0.747–0.858)
≥ 60 years	N/A	N/A	0.667 (0.615–0.719)	0.698 (0.646–0.749)
PCE Risk Categories				
Borderline & Intermediate	N/A	N/A	0.652 (0.594–0.709)	0.688 (0.634–0.742)

## Data Availability

Data used in this manuscript is not publicly available due to prior legal agreements. However, readers may reach out to the corresponding author to receive access to the pooled data source
